# Funding mechanisms as incentive systems: a role for science philanthropy

**DOI:** 10.3389/fsysb.2026.1939099

**Published:** 2026-10-23

**Authors:** Lillian Parr, Nadia Sarfraz, Sylvina M. Raver, Nadia M. Penrod

**Affiliations:** Milken Institute Science Philanthropy Accelerator for Research and Collaboration, Washington, DC, United States

**Keywords:** biomedical research, funding priorities, incentives, philanthropy, scientific research

## Abstract

Biomedical research is shaped not only by scientific questions, but also by the incentive structures that determine what scientists study, how they approach the work, and how success is defined. Current incentives often prioritize individual achievement as a pathway to career advancement, while undervaluing replication, data sharing, mentorship, collaboration, and shared infrastructure. This can make it challenging for scientists to prioritize these contributions, even when they recognize that they are essential for a strong scientific ecosystem. This article examines how funders and funding mechanisms create implicit incentives and argues that funding design is a powerful lever for shaping both scientific behavior and the broader research ecosystem. Philanthropy is uniquely positioned to experiment with funding design by supporting high-risk research, emerging areas, and investments in people, fields, norms, and shared infrastructure that other funders may be unable or unwilling to pursue, while also dedicating resources to learn from how these investments shape scientific behavior. Here, we highlight questions funders can ask about the incentives embedded in funding mechanisms, including what behaviors they encourage, who participates and benefits, and what persists beyond the award. For scientists, understanding funding goals, participating in evaluations, and making contributions beyond traditional outputs visible can strengthen the feedback between funding design and scientific practice. By treating funding mechanisms as design choices that can be tested and refined, philanthropy can generate evidence that informs future funding approaches and strengthens the scientific ecosystem.

## Introduction

Funding mechanisms that support scientific research communicate two things: the science they want to advance and the behaviors they reward. The first is usually made explicit through funding priorities, eligibility criteria, and review criteria. The second is often implicit, emerging through the design of funding mechanisms and the pattern of funding decisions over time, which signal not only what kinds of science are valued but also which researchers and institutions are considered most likely to generate important scientific advances.

Because these signals shape behavior, every funding decision influences the questions researchers ask, the risks they take, how they collaborate, what they share, and which contributions are ultimately recognized (Fortunato et al., 2018). The design choices that shape how investments in science are structured and allocated—collectively referred to as funding design—include the type of funding mechanism, eligibility requirements, decision-making processes, award terms, and evaluation approaches. Funding design is a powerful lever for shaping behavior (Azoulay and Li, 2020).

Scientific progress depends on a diverse funding ecosystem in which different funders bring different priorities, risk tolerances, and time horizons. Public agencies provide foundational support for basic and translational research while operating under requirements for accountability and public oversight. Industry funding plays a critical role in advancing discoveries toward applications, including therapeutics and diagnostics, with decisions shaped by scientific opportunity, unmet needs, development feasibility, and the potential for financial return. Philanthropic funding often supports areas that fall outside traditional funding priorities, including emerging fields, underfunded diseases, and high-risk approaches with uncertain timelines or outcomes (Shekhtman et al., 2024). These funding mechanisms are guided by philanthropic missions and the priorities of individual donors and organizations, often with more flexible accountability structures than public or industry funders. This flexibility enables philanthropy to experiment with funding design—testing how different approaches to selecting, supporting, and evaluating researchers shape scientific behavior and research communities over time, and generating evidence to improve funding mechanisms.

## The incentive structures that shape science

Biomedical research is shaped by more than the quality of ideas. It is also shaped by the systems that determine which behaviors are rewarded and which contributions are recognized. These systems are not neutral. When hiring, promotion, and grant renewal prioritize publications over replication, novelty over rigor, and speed over collaboration, investigators respond rationally to those incentives (Gross and Bergstrom, 2024).

One consequence of these incentive structures is that scientific achievement is often evaluated through a limited set of indicators. Publications, citations, and grant funding are commonly used as indicators of scientific achievement. Awards, journal reputation, and institutional affiliation inform perceptions of status and influence. Yet these signals capture only part of what enables scientific progress. Many researchers already recognize the variety of activities that strengthen and sustain the scientific ecosystem, including replication, data sharing, mentorship, collaboration, and building shared resources, but these contributions often carry less weight in decisions about funding, promotion, and recognition (Rice et al., 2020). So the challenge is not persuading scientists that these activities matter; it is ensuring that the systems governing scientific careers consistently recognize and reward contributions that improve the reliability, efficiency, inclusiveness, and long-term capacity of scientific research.

The signals conveyed through funding decisions also shape broader patterns of prestige and opportunity, influencing which researchers, approaches, and fields receive attention and resources. These dynamics can become self-reinforcing: visibility attracts funding, talent, and recognition, which further concentrates opportunities among already prominent researchers, institutions, and fields (Merton, 1968; Bol et al., 2018). Research on scientific prizes illustrates these dynamics. Prizes not only recognize achievement but also reinforce networks of influence, shaping which researchers and ideas gain visibility and how recognition spreads across scientific communities (Ma and Uzzi, 2018). Researchers may therefore gravitate toward dominant paradigms and well-connected networks, where recognition and collaboration opportunities are more accessible, rather than pursuing ideas that challenge prevailing views or create professional friction with influential figures (Begley, 2019).

## Every funding mechanism creates incentives

Every funding mechanism embodies a different answer to the same fundamental question: What effectively drives scientific progress? Each funding mechanism reflects a different set of assumptions about how to achieve impact, and those assumptions create tradeoffs in what the mechanism prioritizes.

Investigator-based awards assume that talented researchers are best positioned to identify important questions and, by providing flexibility, to pursue them effectively, but selection based on standard indicators of achievement may reinforce existing advantages.

Project-based grants assume that promising ideas can be evaluated through defined research plans, thereby enabling rigorous assessment of proposed work, but may favor incremental advances over bold breakthroughs (Packalen and Bhattacharya, 2020).

Milestone-driven funding assumes that structured goals and accountability improve progress, which can accelerate targeted efforts, but may constrain adaptation when discoveries lead research in unexpected directions.

Prize mechanisms assume that recognition can shape scientific behavior by directing attention and conferring prestige, but their influence depends on which achievements, activities, and scientific values they signal as worthy of recognition.

The mechanisms used to evaluate scientific proposals and investigators also shape incentives. Peer review assumes that expert judgment can identify promising science and effectively allocate resources, but because reviewers evaluate work through the lens of their expertise, experiences, and disciplinary norms, peer review can also introduce or reinforce biases in favor of established approaches, institutions, or research paradigms (Travis and Collins, 1991; Langfeldt, 2006; Teplitskiy et al., 2022).

## Philanthropy can experiment with funding design

Different funding mechanisms are each suited to different goals, which creates opportunities for experimentation. Philanthropic funders can invest both in new funding approaches and in the time, resources, and expertise required to learn whether those approaches achieve their intended effects over time. Systematic evaluation can test how funding design influences researcher behavior and scientific norms: Does flexibility change how researchers approach risk and uncertainty? Do recognition mechanisms influence scientific norms and priorities? Do alternative evaluation approaches change who contributes and which contributions are rewarded and sustained?

The Volkswagen Foundation’s Experiment! Initiative provides one example. Between 2013–2021, the Foundation supplemented expert review with partial randomization, selecting some scientifically vetted, eligible proposals through an interdisciplinary jury and others by lottery (Knoke, 2024). The approach was intended to increase diversity and reduce potential bias in consensus-oriented conventional review, and the Foundation commissioned research to evaluate its effects. The results showed greater diversity among grantees, including higher shares of early-career researchers and women, with jury-selected and lottery-selected projects showing no difference in standard outcome metrics such as the number of publications and patent applications. Participating researchers viewed partial randomization as a way to mitigate conflicts of interest and unconscious bias: 88% of respondents agreed that randomized selection clearly avoids both, offering opportunity to topics and thematic and methodological approaches that may be underrepresented on review panels. This illustrates how philanthropic funders can evaluate funding design to understand how changes affect who and what gets funded, and how the resulting research portfolio performs on standard outcome metrics to build an evidence base that can inform funding practices more broadly.

## Designing funding mechanisms to shape scientific ecosystems

Making behavioral incentives explicit creates opportunities to design funding mechanisms more intentionally, creating conditions in which the behaviors that advance scientific progress become the rational professional choice.

Recognizing that funding decisions shape the research ecosystem, not just individual projects or their immediate outputs, philanthropic investments can reinforce complementary parts of that ecosystem, including people, fields, norms, and shared infrastructure.

### Investing in people

Investment in people shapes how scientists approach research, influencing the questions they pursue, the risks they take, and the time horizons they consider. Long-term, flexible investigator awards can reduce pressure to optimize research agendas for short-term outputs and instead give scientists greater freedom to follow emerging evidence, revise hypotheses, and pursue uncertain but potentially transformative directions.

Howard Hughes Medical Institute’s Investigator program illustrates this approach by providing long-term, flexible support to researchers rather than requiring detailed project plans. Comparative studies suggest that investigators supported through this model pursue more novel research directions and produce a wider distribution of scientific outcomes than similarly accomplished National Institutes of Health (NIH)-funded investigators (Azoulay et al., 2011).

### Investing in fields

Investment in scientific fields shapes the formation, development, and evolution of scientific communities. Support for research networks, evidence synthesis, and other collective scientific efforts can help scientific communities define shared priorities, establish common approaches and standards, accumulate and integrate knowledge, and build the visibility and legitimacy needed to attract researchers and sustain continued investment.

The Chan Zuckerberg Rare As One initiative illustrates this approach by supporting patient-led organizations in building collaborative research networks, strengthening organizational capacity, and aligning researchers, clinicians, and communities around shared priorities in rare disease research. This model invests in the capacity of the scientific community to coordinate research and accelerate progress across fragmented disease areas (Rare As One Network Grantees, 2026).

### Investing in norms

Investment in norms shapes which scientific behaviors become expected, recognized, and rewarded within research communities. These norms influence decisions about what gets shared, how contributions are credited, which forms of evidence are valued, and how researchers are evaluated for hiring, promotion, and funding. Support for open data practices, reproducibility efforts, responsible research assessment, and other shared expectations can help translate aspirational values into routine behavior (Hicks et al., 2015; Moher et al., 2020; Arentoft et al., 2022).

The Centre for Human-Specific Research, which has a mission to advance non-animal research methods, provides one example of how philanthropic funding can expand the methodological approaches available to scientists (Centre for Human Specific Research: Grants and Funding, 2025). The organization funds graduate students and early-career researchers developing and validating non-animal methods. Many scientists feel compelled to incorporate animal models into their research because they are a customary step in their field, or because they anticipate that peer reviewers will request them (Krebs, 2023). This can be the case even when validated alternatives exist or when animal models are not necessary for a particular study (Marshall et al., 2023; Ro, 2025). By supporting researchers who develop and validate alternative approaches, philanthropy can create opportunities to pursue methods that fall outside established scientific norms. Broader efforts to redesign research assessments reflect the same principle. Initiatives including the Declaration on Research Assessment (DORA), the Leiden Manifesto, and the Hong Kong Principles advocate evaluating researchers using a broader range of contributions than publication-based metrics alone (Cagan, 2013; Hicks et al., 2015; Moher et al., 2020). Narrative CV formats represent one approach to implementing these principles, giving researchers space to describe contributions such as mentorship, collaboration, data sharing, methods development, and community resources that are not captured by publication lists alone (Shaw, 2024).

Recognition mechanisms similarly reinforce behaviors that traditional evaluation systems often overlook. The NIH Replication Prize rewards rigorous confirmatory research, the AAAS Mentor Award recognizes sustained mentorship as scientific leadership, and the Parasite and Symbiont Awards celebrate exemplary data sharing and reuse (Announcing the Winners of the NIH Replication Prize: Shaping the Future of Rigorous Science, 2026; AAAS Mentor Awards, 2026; The Parasite Awards, 2026; The Symbiont Awards, 2026).

The Modernizing Academic Appointment and Advancement Challenge extended this approach to institutional evaluation by supporting universities in redesigning hiring and promotion criteria to recognize open science, interdisciplinary collaboration, and mentorship (Modernizing Academic Appointment and Advancement MA3 Challenge, 2025).

### Investing in infrastructure

Investment in shared research infrastructure creates scientific public goods that benefit entire research communities. Funding for data repositories, computational platforms, biobanks, multicenter trial networks, and other shared resources reduces duplication, expands access to research tools, and enables research beyond the capacity of individual investigators or institutions. Because these resources can be shared, reused, and built upon over time, they continue to generate scientific value long after the original investment.

The Gordon and Betty Moore Foundation’s Data-Driven Discovery Initiative illustrates this ecosystem approach. Alongside investments in researchers and academic environments, the initiative supported software tools and other shared resources for data-driven science. An independent evaluation found that these complementary investments reinforced one another and that modest investments in widely shared tools can generate substantial scientific value (Epstein et al., 2017).

### Structuring scientific work

Funding design also shapes how scientific work is organized. Funding mechanisms that support collaborative teams, multidisciplinary networks, and shared research efforts can foster collective problem solving and knowledge integration, whereas mechanisms centered on individual investigators may place greater emphasis on individual achievement and recognition. Neither approach is inherently preferable, but each creates different conditions for how scientific communities generate, share, and build knowledge over time. Wellcome Leap, for example, organizes multidisciplinary teams around ambitious, time-limited scientific challenges, illustrating how funding design can incentivize collaboration, accountability, and urgency (Wellcome Leap, 2026).

Because the scientific ecosystem is an interconnected system, the effects of any investment in people, fields, norms, or infrastructure depend not only on what is funded but also on the incentives and structures that surround it (Tiokhin et al., 2024). Long-term support for researchers is more likely to change behavior when funding mechanisms evaluate and recognize the full range of contributions needed for scientific progress. Investments in shared infrastructure are more valuable when communities establish standards and practices that enable its use, interoperability, and reuse. Collaborative efforts are more sustainable when evaluation systems recognize collective contributions alongside individual achievements.

Designing funding mechanisms requires making trade-offs. Funding mechanisms that encourage researchers to pursue uncertain research directions, collaborate across boundaries, or build shared resources may generate greater long-term value, but at the expense of less predictable short-term outcomes and greater difficulty in attributing accomplishments to individual contributors. Making these trade-offs explicit is essential for aligning funding decisions with the scientific behaviors and outcomes they are intended to support.

## Evaluating funding as an incentive system

Viewing funding through the lens of incentive design changes how investments in science should be evaluated. Because incentive systems influence researcher behavior in complex and evolving ways, even comprehensive evaluation measures provide only a partial picture of their effects. A funding mechanism can achieve the results it was designed to measure while also creating unintended incentives that influence researcher behavior, collaboration patterns, and scientific priorities over time. Evaluation should therefore examine both whether a funding mechanism achieves its intended outputs and how its underlying incentives shape researcher behavior and whether those changes produce intended or unintended effects. Funders can, for example, track changes in research practices and collaboration, compare outcomes across funding mechanisms or before and after changes in funding design, and use surveys, interviews, and administrative data to understand how funding conditions influence research decisions. For funding designed to encourage higher-risk research, evaluation might compare whether recipients pursue more novel research than comparable researchers receiving conventional funding, and whether those changes persist over time. No single measure can show that a funding mechanism caused a change in behavior, but combining multiple sources of evidence can help funders assess whether a mechanism is producing the intended behaviors and effects, and identify unintended consequences, across the scientific ecosystem. This evidence can then improve future funding mechanisms and decisions. A useful framework asks three questions: What behaviors and norms does the funding mechanism encourage? Who participates and benefits? What persists beyond the award?

### What behaviors and norms does the funding mechanism encourage?

Over time, researchers adapt to funding mechanisms and this can shift or reinforce broader community expectations about how science should be conducted. Relevant questions include whether mechanisms encourage collaboration, openness, knowledge sharing, mentorship, methodological rigor, and calculated risk-taking, or whether they reinforce narrower incentives around individual productivity and short-term outputs. These effects can be evaluated through quantitative and qualitative evidence, including changes in data sharing, collaboration, replication, open research practices, and contributions to shared resources. For a mechanism designed to encourage collaboration, for example, evaluate might consider whether recipients from more cross-disciplinary or new collaborations than comparable researchers and whether those relationships persist beyond the period of funding. Surveys and interviews can complement these measures by revealing how funding mechanisms influence researcher decision-making, and comparisons across funding mechanisms can help identify how funding design may influence behavior. The relevant outcomes are therefore not only research products, but also shifts in the behaviors and practices that enable sustained scientific progress.

### Who participates and benefits?

Funding mechanisms influence which researchers, institutions, and communities gain opportunities to contribute, develop expertise, and shape scientific priorities. Relevant questions include whether funding mechanisms expand participation, support researchers at different career stages, create opportunities for institutions with fewer existing resources, or concentrate resources among groups that are already well positioned to succeed. These effects can be assessed by evaluating who receives funding, who participates in funded activities, and what opportunities follow from that participation. Useful measures include the distribution of awards across career stages and institutions, the proportion of first-time recipients, and the concentration of funding among a small number of institutions. For example, for a mechanism intended to broaden participation, evaluation might consider whether it attracts more first-time applicants or recipients from less-funded institutions than conventional funding mechanisms, and whether participation leads to subsequent research opportunities. Surveys and interviews can help explain why specific groups participate or do not participate, including whether eligibility requirements, review criteria, or application processes create barriers to entry. Such evidence can reveal whether a funding mechanism expands scientific capacity by bringing a broader range of researchers, institutions, perspectives, and approaches into scientific discovery, or reinforces existing concentrations of resources and opportunity.

### What conditions persist beyond the award?

The value of many investments in science lies not only in the immediate activities they support, but in the communities, tools, datasets, standards, and infrastructure they create for future work. Relevant questions include whether these assets are shared and reused, whether collaborations continue, whether improved practices become embedded in scientific norms, and whether investments create lasting capabilities that enable future scientific discovery. These effects can be evaluated by tracking whether resources created through an award continue to be used, maintained, or built upon after funding ends; whether collaborations generate subsequent joint work; whether practices introduced through a funding program are adopted beyond the original recipients; and whether communities or infrastructure develop new sources of support. For an investment intended to build shared research capacity, for example, evaluation might consider whether datasets or tools are actively used after the award period and whether they generate new research or collaborations beyond the original grantees. These longer-term effects are central to understanding the full value of investments designed to strengthen the scientific ecosystem, helping distinguish outputs produced during a funding period from capabilities that continue to generate value after the original investment.

Evaluation should be built into the design of funding mechanisms, not added after funding decisions have been made. Eligibility requirements, review criteria, award terms, reporting expectations, and renewal decisions all create incentives that shape researcher behavior. Funders who measure success primarily through publications, citations, patents, and follow-on funding will encourage researchers to optimize for those measures, regardless of how their funding mechanisms are designed. Many important scientific contributions—including datasets, reusable software, methods, negative results, and community resources—are only weakly reflected in traditional publication-based metrics despite their long-term value to scientific progress (Hicks et al., 2015). Building evaluation into funding design allows funders to identify which contributions their measures capture, which they overlook, and whether incentives align with the behaviors needed to advance scientific progress.

Evaluation can also serve as a mechanism for learning, not merely accountability. Philanthropy has the flexibility to test new approaches, adapt funding mechanisms as evidence emerges, and share lessons about which funding mechanisms best advance scientific progress. This is particularly important when funding mechanisms produce unintended effects that may only become visible over time. For example, the Howard Hughes Medical Institute revised eligibility for its 2026 Investigator Program after identifying overrepresentation from a small number of institutions among previous awardees, illustrating how evaluating patterns in funding can identify unintended consequences and inform subsequent changes to funding design (Mervis, 2024).

Finally, evaluation practices themselves shape incentives. The measures funders choose to assess progress signal what activities are valued and rewarded (Hicks et al., 2015; Arentoft et al., 2022). Evaluation approaches that recognize collaboration, methodological rigor, knowledge reuse, mentorship, and contributions to shared scientific resources can reinforce the behaviors needed for a productive and resilient research ecosystem.

Scientists also have a role to play in improving funding mechanisms. Researchers can understand what a funding mechanism is designed to support, including behaviors, contributions, and outcomes that funders intend to encourage. They can participate in evaluations and provide feedback on whether funding conditions enable those goals in practice, and use features of the funding mechanism as intended, such as flexibility to pursue unexpected findings or support collaboration and shared resources. Researchers can make less visible contributions visible by documenting activities such as mentorship, data sharing, replication, methods development, and collaboration, and can provide information about unintended incentives when eligibility requirements, reporting expectations, or evaluation criteria create barriers or encourage behaviors that differ from the funder’s intent. Together, these actions can give funders the information needed to refine funding mechanisms over time.

## Conclusion

Science philanthropy has historically been viewed primarily as a funding source for research. Its deeper opportunity is to become a source of learning about which approaches to funding, evaluation, and recognition best support scientific progress. Funding is not neutral: it shapes incentives, behaviors, and scientific norms. By treating funding mechanisms as design choices that can be tested and refined, philanthropy can help build the evidence base needed to strengthen a scientific ecosystem that is more reliable, creative, and capable of generating knowledge as a public good.

## Data Availability

No publicly available datasets were analyzed in this study.

## Author's note

The Milken Institute Science Philanthropy Accelerator for Research and Collaboration (SPARC) works with philanthropists and nonprofit organizations to create high-impact initiatives that accelerate biomedical research and turn innovation into real-world health improvements. Through our work, we actively work to build a bridge between the bench and the bedside by working with researchers, patient advocates, and funders.
